# Mitomycin-treated undifferentiated embryonic stem cells as a safe and effective therapeutic strategy in a mouse model of Parkinson’s disease

**DOI:** 10.3389/fncel.2015.00097

**Published:** 2015-04-08

**Authors:** Mariana Acquarone, Thiago M. de Melo, Fernanda Meireles, Jordano Brito-Moreira, Gabriel Oliveira, Sergio T. Ferreira, Newton G. Castro, Fernanda Tovar-Moll, Jean-Christophe Houzel, Stevens K. Rehen

**Affiliations:** ^1^Institute of Biomedical Sciences, Federal University of Rio de JaneiroRio de Janeiro, Brazil; ^2^D’Or Institute for Research and Education (IDOR)Rio de Janeiro, Brazil; ^3^Institute of Medical Biochemistry Leopoldo de Meis, Federal University of Rio de JaneiroRio de Janeiro, Brazil; ^4^Oswaldo Cruz Institute, Oswaldo Cruz FoundationRio de Janeiro, Brazil

**Keywords:** embryonic stem cell, stem cell therapy, Parkinson model, mitomycin C, contrast-enhanced MRI

## Abstract

Parkinson’s disease (PD) is an incurable progressive neurodegenerative disorder. Clinical presentation of PD stems largely from the loss of dopaminergic neurons in the nigrostriatal dopaminergic pathway, motivating experimental strategies of replacement based on cell therapy. Transplantation of dopaminergic neurons derived from embryonic stem cells significantly improves motor functions in rodent and non-human primate models of PD. However, protocols to generate dopaminergic neurons from embryonic stem cells generally meet with low efficacy and high risk of teratoma formation upon transplantation. To address these issues, we have pre-treated undifferentiated mouse embryonic stem cells (mESCs) with the DNA alkylating agent mitomycin C (MMC) before transplantation. MMC treatment of cultures prevented tumorigenesis in a 12 week follow-up after mESCs were injected in nude mice. In 6-OH-dopamine-lesioned mice, intrastriatal injection of MMC-treated mESCs markedly improved motor function without tumor formation for as long as 15 months. Furthermore, we show that halting mitotic activity of undifferentiated mESCs induces a four-fold increase in dopamine release following *in vitro* differentiation. Our findings indicate that treating mESCs with MMC prior to intrastriatal transplant is an effective to strategy that could be further investigated as a novel alternative for treatment of PD.

## Introduction

Parkinson’s disease (PD) is an incurable progressive neurodegenerative disorder with devastating consequences for patients and their families (Paulsen et al., 2013). Both genetic and environmental elements play a role in its etiology, with aging as the major risk factor. As the elderly population rises around the world, the number of affected individuals increases accordingly (Lees et al., 2009). Pharmacological treatments provide adequate symptomatic relief for many patients in the short term; however, severe adverse effects develop over time and drugs fail to prevent disease progression (Obeso et al., 2010).

Clinical presentation of PD stems largely from the loss of dopaminergic neurons in the nigrostriatal pathway. Many experimental strategies have therefore aimed to replace these cells. In humans, transplantation of dopamine (DA) neurons derived from fetal ventral mesencephalon has obtained clinical success (Piccini et al., 1999; Freed et al., 2001), but use of such neurons remains ethically controversial, besides the fact that resources are limited.

Mesenchymal stem cells have been suggested as a potential alternative source to fetal neurons (Khoo et al., 2011; White, 2011). However, they fail to readily differentiate into dopaminergic neurons, which has diminished the enthusiasm for using such cells for PD therapy. Embryonic stem cells retain the capacity to differentiate into DA neurons, both *in vitro* and *in vivo*. Importantly, transplantation of embryonic stem cell-derived DA neurons significantly improves motor functions in rodent and non-human primate models of PD (Deacon et al., 1998; Bjorklund et al., 2002; Muramatsu et al., 2009; Arenas, 2010; Kriks et al., 2011).

Several protocols have been described for differentiation of embryonic stem cells into dopaminergic neurons, albeit with low efficacy (Kirkeby et al., 2012; Sundberg et al., 2013). In addition, the resulting cell population often contains serotoninergic neurons, as well as residual undifferentiated embryonic cells that have a strong potential to give rise to teratomas, thereby limiting the clinical applicability of such cultures for transplantation into patients (Kriks and Studer, 2009; Chung et al., 2011; Lindvall, 2012). Moreover, published protocols are generally expensive and time consuming, and fail to yield large amounts of differentiated neurons, as required for clinical trials. Thus, there is an urgent need for alternative strategies that are safe and capable of generating purified embryonic stem cell-derived DA neurons on a large scale.

In order to minimize technical issues and eliminate the tumorigenic risk of transplanted cells, we tested for the first time the use of fully undifferentiated embryonic stem cells treated with the DNA alkylating and crosslinking agent mitomycin C (MMC) as an alternative source for cell transplant in a PD model. MMC is an FDA (2002)-approved chemotherapeutic agent for pancreatic and gastric adenocarcinoma that has also been tested in combination with other drugs in a wide variety of solid tumors (Akhlaghpoor et al., 2012; Werner et al., 2013). Our findings indicate that transplantation of undifferentiated embryonic stem cells pre-treated with MMC is a safe and effective strategy, and should be further investigated as a novel alternative for treatment of PD.

## Materials and Methods

### Animals

All experiments were performed in accordance with International Guidelines for the Care and Use of Mammals in Neuroscience and Behavioral Research, and all procedures were approved by the University Committee on Ethics of Animal Use (protocol #DAHEICB060 for meningeal cell primary culture and for stem cell transplantation, and #DAHEICB027 for teratoma assay and 6-OHDA lesion). Mice were housed in self-ventilated cages (6 per cage) under standard laboratory conditions (12 h light/dark cycle, food and water *ad libitum*).

### Embryonic Stem Cell Culture

USP1 mouse embryonic stem cells (mESCs) transfected with enhanced green fluorescent protein (eGFP) with retroviral vector (Paulsen et al., 2011) were maintained on a cell-free murine embryonic fibroblast-based substrate to avoid fibroblast contamination between cell passages (Stelling et al., 2013). Cells were maintained in D-MEM/F12 (Gibco) supplemented with 15% fetal bovine serum (JRH Biosciences), 2 mM glutamine (Gibco), 0.1 mM non-essential amino acids (Gibco), 0.1 mM β-mercaptoethanol (Gibco), 40 µg/mL gentamicin sulfate (Schering-Plough), and 0.2% of conditioned medium of CHO (Chinese hamster ovary) cells producing leukemia inhibitory factor (LIF).

### Mitomycin C Treatment and Apoptosis Assay

For mitotic inactivation, undifferentiated mESC colonies were treated for 12 h with different concentrations of MMC (1, 2.5, 5, or 10 µg/mL). Immediately after MMC treatment, apoptosis was measured by the number of caspase 3 positive cells by flow cytometry. After incubation with MMC, mESCs were washed three times with D-MEM/F12, dissociated from the substrate into a single-cell suspension with 0.25% trypsin-EDTA (Gibco-Invitrogen) for 2 min, fixed with 4% paraformaldehyde in PBS for 20 min, and permeabilized with 0.3% Triton X-100 for 5 min. Cells were then blocked with 5% bovine serum albumin (BSA) solution for 30 min and incubated for 3 h with primary anti-mouse antibody against the active (cleaved) form of caspase 3 (Millipore), 1:400 in 1% BSA solution. Antibody-antigen reaction was revealed by a secondary antibody conjugated to Alexa Fluor 546, 1:1000 (Invitrogen). Acquisition was performed using a fluorescence-activated cell sorting (FACS) Calibur flow cytometer (Becton-Dickinson, USA) and data analysis was performed using WinMDI 2.8 software. A total of 100,000 events were acquired from three independent experiments for each tested MMC concentration.

### *In Vitro* Neural Differentiation Over Meningeal Cells

Dopaminergic differentiation was induced as described in Hayashi et al. (2008). Meningeal cell cultures were prepared from postnatal day 0 C57BL/6 mice. Briefly, the meninges were dissected from the calvaria and cultivated in α-minimum essential medium (MEMµ; Gibco-Invitrogen) containing 10% fetal bovine serum (JRH Biosciences), penicillin G (40 U/mL), and streptomycin (50 µg/mL). After the first passage, meningeal cells were allowed to reach confluence and were used as a feeder layer for mESCs. Mitomycin-treated and non-treated mESC colonies were dissociated with 0.25% trypsin-EDTA (Gibco-Invitrogen) for 2 min and plated on confluent meningeal layers (200 cells/cm^2^). Co-cultures were maintained for 14 days in differentiation medium: G-MEM (Gibco-Invitrogen) supplemented with 5% knockout serum replacement (Gibco-Invitrogen), 2 mM glutamine, 0.1 mM non-essential amino acids, 1 mM pyruvate, and 0.1 mM β-mercaptoethanol.

### Electrophysiological Recordings

Voltage-clamp recordings were made from neuron-like cells 14 days after co-culturing control or mitomycin-treated mESCs eGFP positive cells on top of the meningeal cell layer. Whole-cell currents were recorded through borosilicate glass microelectrodes (WPI, USA) prepared on a P-97 horizontal puller (Sutter Instruments, USA). Current signals were acquired with an EPC-7 (HEKA, Germany) amplifier, low-pass filtered at 1 kHz, and digitized at 10 kHz with a LabMaster interface under the control of pClamp software (Axon Instruments, USA). Extracellular recording solution contained (in mM): 165 NaCl, 5 KCl, 2 CaCl_2_, 10 dextrose, 5 HEPES, 2 NaOH, pH 7.35. Cells were perfused at the rate of 1 mL/min at room temperature (23°C) throughout the recordings. The microelectrode (intracellular) solution included (in mM): 80 CsCl, 80 CsF, 10 EGTA, 10 HEPES, and 26 CsOH, pH 7.30. Filled patch microelectrodes had resistances ranging from 2.7 to 7.4 MΩ when measured in the bath and a −7 mV liquid junction potential was added to the reported clamp potentials. Membrane potential was held at −70 mV and, approximately 2 min after achieving the whole-cell configuration, voltage-sensitive currents were evoked by 200 ms depolarizing square pulses ascending in 10 mV steps from −60 to +60 mV, preceded by a 10 ms hyperpolarization to −90 mV. Leak-subtracted current traces were obtained by the fractional method (P/4) using four scaled hyperpolarizing subpulses. Data were analyzed using Clampfit 9 software (Axon Instruments, USA).

### Immunofluorescence for Dopaminergic Neurons

Following 2 weeks of neuronal differentiation, co-cultures were fixed with 4% paraformaldehyde in PBS for 20 min, permeabilized with 0.5% Triton X-100 for 5 min, blocked with 5% BSA for 60 min, and incubated overnight at 4°C with the primary antibodies mouse β-tubulin III (1:400, Sigma), or rabbit anti-tyrosine hydroxylase (1:500, Millipore). Antibody-antigen reaction was visualized using anti-rabbit Alexa-594- or anti-mouse Alexa-594-coupled secondary antibodies (1:1000, Invitrogen). Cell cultures were analyzed and images were captured using a TCS SP5 laser confocal microscope (Leica Microsystems).

### Dopamine Release Assay

The amount of dopamine released spontaneously by mESC cells into the conditioned medium for 48 h, or after stimulation by elevated KCl solution, was measured by reverse phase chromatography coupled with electrochemical detection (0.5 V), as previously described (Arita et al., 2002). Cells were washed twice in a low KCl solution (in mM: HEPES-NaOH, 20, pH 7.4; NaCl, 140; KCl, 4.7; CaCl_2_, 2.5; MgSO_4_,1.2; KH_2_PO_4_, 1.2; glucose, 11) and incubated for 2 min in the same solution. Cells were then incubated for 15 min in a high KCl solution (in mM: HEPES-NaOH, 20, pH 7.4; NaCl, 85; KCl, 60; CaCl_2_, 2.5; MgSO_4_, 1.2; KH_2_PO_4_, 1.2; glucose, 11) to promote membrane depolarization. Briefly, 2 mL aliquots of culture medium and 0.8 mL of low or high KCl solutions, from control or mitomycin-treated differentiated mESCs, were first submitted to the following purification steps: 50 mg alumina (Al_2_O_5_) was weighed out in centrifuge tubes and the samples were added to 1 mL Tris-buffer, pH 8.0, 103 mM EDTA, plus 3 µL of 1 mM dihydroxybenzylamine (DHBA, internal standard). The suspension was mixed for 10 min at 4°C, protected from light. Precipitated alumina was washed three times with 1 mL of ultrapure water and dopamine was eluted with 400 mL of 100 mM perchloric acid after 3 min of vortex agitation. After centrifugation for 3 min at 1,000 × g, 100 µL of medium and supernatant were analyzed. Isocratic separation was obtained using a C18 reverse phase column (Supelco 4.6 × 250 mm, Sigma Aldrich) eluted with the following mobile phase: 20 mM sodium dibasic phosphate, 20 mM citric acid, pH 2.64, containing 10% methanol, 0.12 mM Na_2_EDTA, and 566 mg/L heptanesulfonic acid. Total time for sample analysis was 30 min. Dopamine concentration was expressed as pg/g cell protein. Pierce BCA Protein Assay Kit was used for total protein quantification.

### Teratoma Assay

To confirm the pluripotency of mESCs and the effectiveness of the mitotic inactivation, mitomycin-treated or non-treated undifferentiated mESCs (500,000 cells) were injected into the thigh muscles of two-month-old nude immunodeficient mice (six animals). After mitomycin treatment, mESC colonies were washed three times with D-MEM/F12, dissociated from the substrate into a single-cell suspension with 0.25% trypsin-EDTA (Gibco-Invitrogen) for 2 min, and suspended in 400 µL of D-MEM/F-12. Using 27 gauge needles (Hamilton), the left upper thigh was injected with control mESCs (non-treated) and the right thigh with mitomycin-treated mESCs (500,000 cells in 2 µL). Animals were continuously monitored. Analgesic was added to the water bottle in order to minimize pain and discomfort (ibuprofen suspension, Abbott, 164 mg/L of drinking water). Twelve weeks after injection, they were anesthetized and submitted to magnetic resonance imaging (MRI, see below), then sacrificed to allow for muscle dissection. Muscles from both hindlimbs were fixed with 5% formalin, embedded in paraffin, and stained with hematoxylin and eosin (HE) for histopathological evaluation.

### Magnetic Resonance Imaging of Teratomas and Transplanted Animals

MRI was performed every 2 weeks after intrastriatal cell injections in all experimental groups. Mice were premedicated with atropine (0.2 mg/kg, s.c.) to reduce vagal tone and then anesthetized with an intraperitoneal injection of ketamine (90–120 mg/kg) and xylazine (10 mg/kg). Images were acquired in a 7-T magnetic resonance scanner (MRI System 7T/210 ASR Horizontal Bore Magnet, Agilent Technologies). Brain images were obtained using proton density (TR/TE: 10/2000 ms; matrix: 128 × 128; slice thickness: 1 mm; no gap, 12 averages), T1-weighted (TE/TR: 15/250 ms; matrix: 128 × 128; slice thickness: 1 mm; no gap, 16 averages), and T2-weighted (TE/TR: 15/2563 ms; matrix: 128 × 128; slice thickness: 1 mm; no gap, 14 averages) sequences in the axial, coronal, and sagittal planes, before and after gadolinium injection (0.2 mL/Kg i.p.). Hindlimb images were obtained using proton density (TR/TE: 10/2000 ms; matrix: 128 × 128; slice thickness: 1 mm; no gap, 16 averages), T1-weighted (TE/TR: 15/800 ms; matrix: 128 × 128; slice thickness: 1 mm; no gap, 5 averages), and T2-weighted (TE/TR: 15/2563 ms; matrix: 128 × 128; slice thickness: 1 mm; no gap, 14 averages sequences) in the axial, coronal, and sagittal planes, before and after injection of 0.2 mL/kg gadolinium (Mesentier-Louro et al., 2014).

Prior to image analysis, datasets were anonymized and randomized across groups. Brain and hindlimb morphology and tumor characteristics were evaluated in blinded fashion by two experienced researchers and compared across groups. For each dataset, all images were visually inspected for artifacts. Data processing was performed using Osirix Software.^1^

#### 6-OHDA-Induced Lesion and Transplantation of mESCs

Neurotoxic injections into the dorsal striatum were carried out according to a previously described protocol (da Conceição et al., 2010). Briefly, after premedicated with atropine (0.2 mg/kg, s.c.) and anesthetized with ketamine (90–120 mg/kg, i.p.) and xylazine (10 mg/kg, i.p.), male Swiss mice received one unilateral injection (2 µL) of 10 µg 6-OHDA (Sigma-Aldrich) dissolved in 0.9% sterile NaCl containing 0.1% ascorbic acid, at the rate of 0.5 µL/min (injection coordinates with respect to bregma were: AP = +0.5 mm; L = −2.0 mm; DV = −3.0 mm). Following the lesion, animals were allowed to recover for 4 weeks prior to an initial behavioral assessment. After additional 4 weeks, mice were re-anesthetized and 500,000 mitomycin-treated or non-treated undifferentiated mESCs, suspended in 2 µL of D-MEM/F12, were transplanted at the same coordinates. Lesion-control animals were injected with culture medium only.

#### Behavioral Analysis

Mice from all experimental groups: lesion-control (*n* = 8), untreated mESCs (mESC, *n* = 8), and mitomycin-treated mESCs (MMC-mESC, *n* = 12), were behaviorally assessed every second week, until 12 weeks after cell transplantation. The behavioral paradigms employed were open field, cylinder, balance beam, grip strength, and apomorphine-induced rotation tests, tested in that order.

Spontaneous activity in the open field was evaluated with the video-tracking EthoVision XT6 system (Noldus Information Technology). After a habituation period, the mice were placed in a 60 × 40 cm arena and locomotor activity, defined as the total covered distance (cm), was evaluated for 5 min. The video was recorded with a camera placed 1.0 m above the observation arena.

Motor coordination deficits were measured by the number of missteps and the latency to cross a 50 cm long, 2.5 cm in diameter round wooden beam. All mice were pre-trained on the beam for 5 min in order to encourage rapid crossing without reversals or stopping. Immediately after pre-training, animals were assessed on the test beam.

Forelimb grip strength was assessed with a grip strength meter with 1 g resolution (Insight EFF305, Brazil). Briefly, the mouse was allowed to grab a bar attached to a force transducer as it was gently pulled by the tail horizontally away from the bar. Force values from three consecutive trials with 5 s intervals were averaged to determine grip strength for each mouse.

The cylinder test was performed as a measure of spontaneous forelimb use (Schallert et al., 2000). Animals were placed individually in a glass cylinder (11 cm diameter, 20 cm height) and examined for spontaneous forepaw contact with the cylinder wall. After each trial, the cylinder was thoroughly cleaned with 70% ethanol. No habituation was allowed until a total of 20 contacts were recorded per animal and only weight-bearing wall contacts made by each forelimb on the cylinder wall were scored. Data are presented as the percentage of lesion/grafted forepaw contacts relative to the total number of contacts, such that symmetric paw use would be scored as 50% (10/20 contacts).

To investigate dysfunction in dopaminergic signaling, analysis of apomorphine-induced rotation was performed as described previously (da Conceição et al., 2010). Apomorphine (0.5 mg/kg) was injected subcutaneously in the scruff and mice were placed in an opaque cylinder 30 cm in diameter. After a 5 min period, full body rotations were manually recorded over a period of 30 min, and data were expressed as net full body turns per minute. Experimental groups were unknown for the observer.

#### Histological Analysis

Four months after cell transplantation, mice were deeply anesthetized and transcardially perfused with PBS followed by 4% PFA. Brains were post-fixed in 4% PFA for 24 h before immersion in a standard sucrose gradient (10%, 20%, and 30%) until they sank. Brains were then embedded in OCT medium (Tissue-Tek). Serial coronal 60 µm thick sections were cut frozen on a cryostat (Leica) and collected in PBS. Sections through the substantia nigra and striatum were processed for immunohistochemistry. Sections were washed with PBS, permeabilized in 1% Triton X-100 for 30 min, incubated for 2 h with 3% H_2_O_2_ diluted in methanol, blocked with 10% BSA for 3 h, and incubated overnight at 4°C with rabbit anti-TH primary antibody, 1:1000 (Millipore). After PBS washing, sections were incubated for 2 h with biotinylated anti-rabbit secondary antibody (Vector Labs, 1:200) and staining was developed using ABC (Vectastain kit) and DAB (Sigma) reactions. Neurons displaying a TH-positive cytoplasm were counted as positive, and plotted throughout the substantia nigra and the striatum using a motorized Zeiss Axioplan microscope and the Neurolucida system (MBF Bioscience).

#### Statistical Analysis

**Figure 1:**
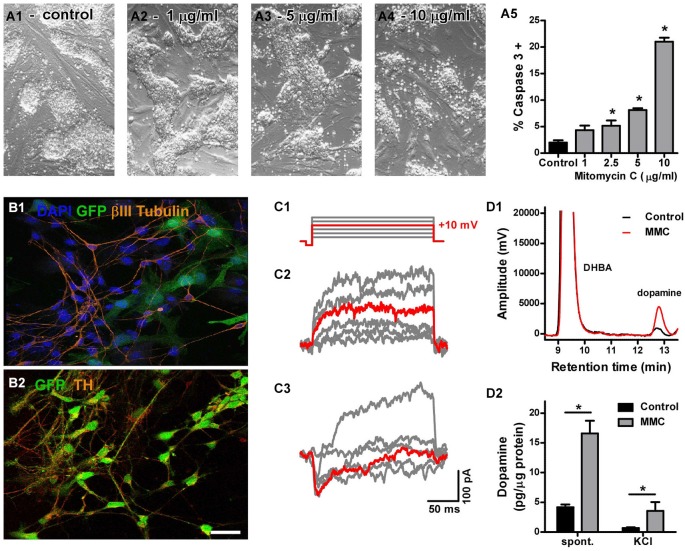
**(A)** Cytotoxicity of mitomycin C (MMC). Phase-contrast micrographs showing the general morphology of control **(A1)** and MMC-treated mESC colonies **(A2–4)**. Cell death induced by treatment with increasing MMC concentrations for 12 h **(A5)** was measured by flow cytometry as the percentage of caspase 3 positive cells. At 1 µg/mL, the effect of MMC was not significant (*n* = 3). **(B)** Assessment of neural differentiation *in vitro*: after 14 days of co-culture onto meningeal cells, most GFP-mESCs (green) pre-treated with MMC (1 µg/mL) became immunoreactive for β-tub III (**B1**, red) and TH (**B2**, red), as expected for dopaminergic neurons. Scale bar: 20 µm. **(C)** Voltage-activated currents from two neuron-shaped mESCs pre-treated with MMC and differentiated for 14 days. Only the steps to −50, −30, −10, +10 (highlighted in red), +30 and +50 mV are shown, for clarity. Most cells showed only outward currents **(C2)** in response to depolarizing voltage steps **(C1)**. One cell in 29 showed inactivating inward currents **(C3). (D1)** Representative chromatogram of culture medium showing the dopamine peak, which increased with MMC treatment, and the DHBA peak (internal standard). **(D2)** Differentiated mESCs pre-treated with MMC released more dopamine to the culture medium spontaneously over 48 h, as well as after 15 min incubation in 60 mM KCl, compared to untreated cultures (* denotes *p* = 0.002 and *p* = 0.013, respectively; Mann-Whitney’s test, *n* = 6).

Numerical values and error bars are expressed as mean ± SEM and mean ± SD for *in vitro* and *in vivo* measurements, respectively. Tests for differences between means were performed using a standard software package (GraphPad Prism version 5.00), using a 5% significance level. In the ANOVA procedures, Dunnet and Tukey tests were used for group comparisons (Figures 1A, 4B–F, respectively). For the apomorphine-induced rotations (Figure 4A), treatments were compared at each time point by a Bonferroni test of inter-subject differences in a repeated measures ANOVA procedure.

**Figure 2:**
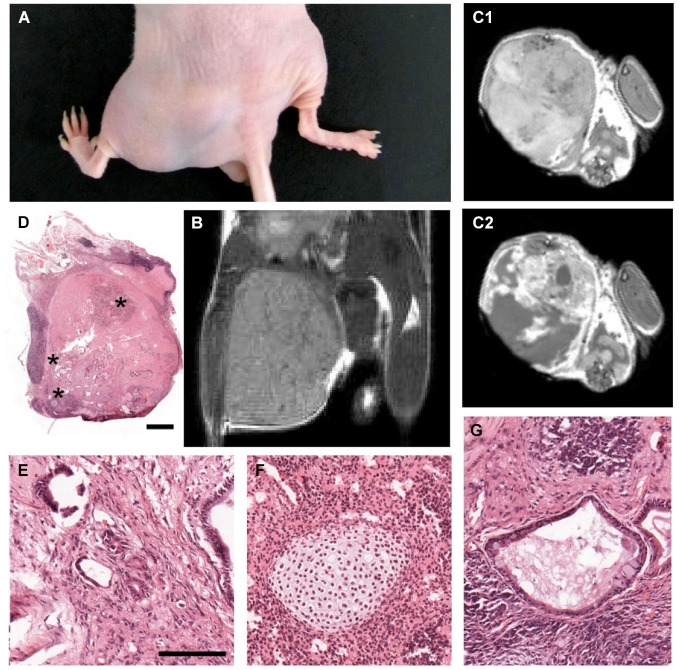
**Tumorigenicity assay in nude mice confirms efficacy of MMC treatment. (A)** Photograph of the hindlimbs from a nude mouse 12 weeks after intramuscular injection of 500,000 mitomycin-treated (right) and untreated (left) mESCs. In all six animals tested, the right limbs were normal and the left limbs displayed large tumors. **(B)** *In vivo* MRI scans, coronal plane, proton density sequence, showing teratoma formation in the left limb, as opposed to the right limb (which received mitomycin-treated mESCs) of the same mouse. **(C)** *In vivo* MRI scans, axial plane, T1-weighted sequence, showing enhancement of gadolinium-contrast signal **(C2)** in the teratoma region. **(D)** High resolution photomontage of hematoxylin-eosin-stained section through the leg injected with untreated mESCs, showing teratoma containing a mixture of tissue types, including: **(E)** neural tube-like, **(F)** cartilaginous, and **(G)** glandular-like (asterisks in **(D)** indicate enlarged regions in **(E**–**G)**). Scale bar: 1000 µm for **(D)**; 100 µm for **(E–G)**.

**Figure 3:**
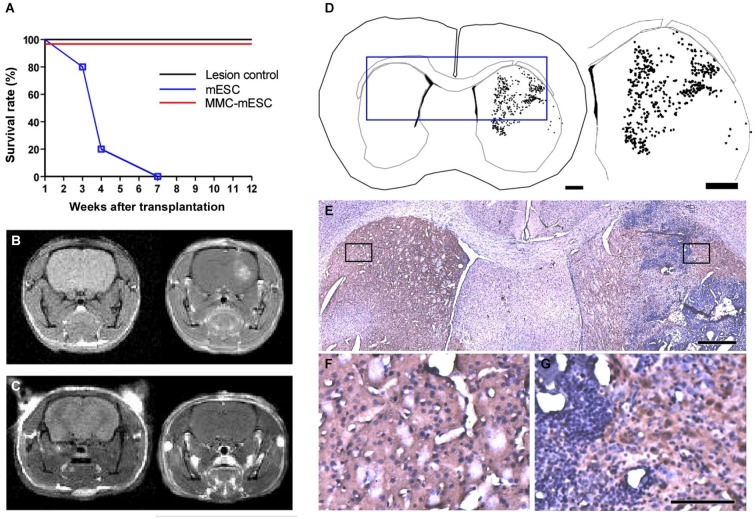
**Transplantation of fully undifferentiated mESCs previously treated or non-treated with MMC into 6-OHDA-lesioned mice brains. (A)** Survival curve of control and transplanted 6-OHDA-lesioned mice: 4 weeks after 6-OHDA injection in the striatum, mice were injected at the same stereotaxic coordinates with culture medium (lesion-control, *n* = 8), untreated mESCs (mESC, *n* = 8), or mitomycin-treated mESCs (MMC- mESC, *n* = 12). **(B,C)** *In vivo* brain MRI, coronal plane, T1-weighted sequence, prior to (left-hand panel) and after (right-hand panels) gadolinium injection evidencing striatal tumor 4 weeks after transplantation of untreated mESCs **(B)** and showing no signs of tumor formation in an animal transplanted with 500,000 mitomycin-treated mESCs for as long as 60 weeks **(C). (D)** Computer-microscope reconstruction of a coronal section through the forebrain of a 6-OHDA-lesioned mouse 4 weeks after transplantation with untreated mESCs into the striatum (dots indicate cell bodies positive for TH-immunostaining). **(E)** Histology of the boxed area in the computer-generated plot in **(D)**; boxed areas in E are shown at higher magnification **(F,G). (F)** non-lesioned, non-transplanted side showing control level of TH staining (brown) in the striatum. **(G)** 6-OHDA-lesioned and mESC-grafted hemisphere, illustrating TH-positive cell bodies within the host striatum in close proximity to the tumor edges. Scale bar: 1000 µm and 1800 µm, respectively, for **(D)**, 500 µm for **(E)**, 100 µm for **(F,G)**.

**Figure 4:**
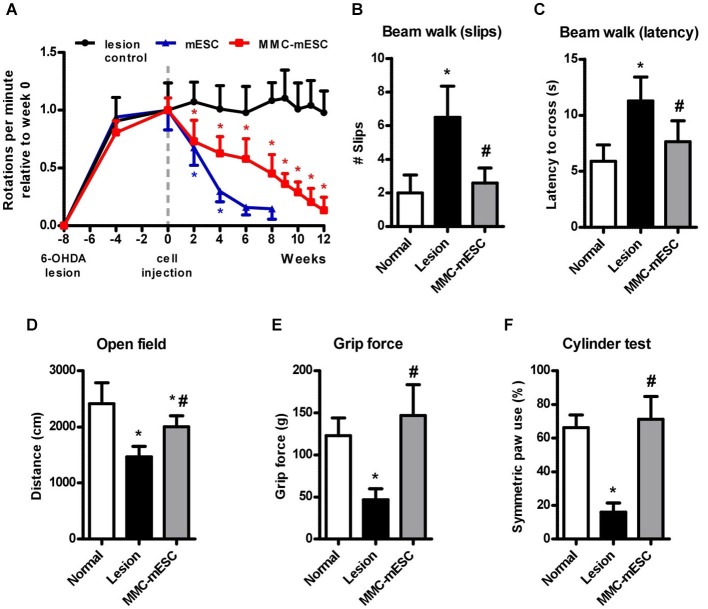
**Functional recovery after transplantation of fully undifferentiated mESCs treated with MMC. (A)** Rotational behavior induced by apomorphine (0.5 mg/kg i.p.) in 6-OHDA-lesioned mice. Eight weeks after the 6-OHDA lesion, mice received intra-striatal injections of culture medium (lesion-control, black), untreated 500,000 mESCs (blue), or 500,000 mitomycin-treated mESCs (MMC-mESC, red). After 12 weeks, all animals transplanted with mitomycin-treated mESCs were alive and had greatly reduced signs of striatal lesion, approaching the performances of normal healthy animals in the different tasks. Motor coordination on the balance beam test was assessed by the number of slips **(B)** and the latency to cross **(C)** a 50 cm long beam. **(D)** Spontaneous locomotor activity in the open field was tracked and expressed as the distance covered within 5 min. **(E)** Forelimb grip strength was measured as the maximal pull force on a bar. **(F)** Forelimb use during vertical exploratory behavior was measured in the cylinder test, in which normal animals most often touched the cylinder wall with both forepaws when rearing. Values are means ± SD. In **(A)**, (*) denotes *p* < 0.01 vs. lesion control; in **(B–F)** (*) denotes *p* < 0.01 vs. normal and ^(#)^ denotes *p* < 0.01 vs. lesion controls in post-ANOVA tests.

## Results

### Treatment with Mitomycin C Increased Dopaminergic Differentiation *in Vitro*

**Figure 5:**
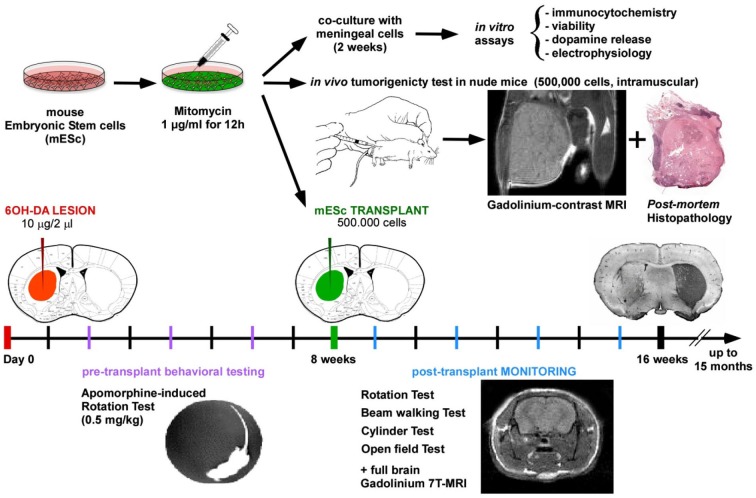
**Schematical summary of the research approach**.

We first determined the optimal concentration of MMC by testing different concentrations *in vitro* and comparing cell death rates to those observed in control cultures. We found that 1 µg/mL MMC was the maximal concentration of mitomycin that did not induce apoptotic cell death compared to control, untreated mESC cultures. Cultures treated with 1 µg/mL MMC for 12 h were morphologically indistinguishable from untreated controls (Figures 1A–5).

We next examined the effect of MMC pre-treatment (1 µg/mL) on the ability of mESCs to differentiate into neurons *in vitro*. After 14 days of co-culture with meningeal cells in conditions promoting dopaminergic differentiation, many MMC-treated mESCs developed neuron-like morphology, and were immunoreactive for β-tubulin III (Figure 1B) and tyrosine hydroxylase (Figure 1B). We first sought to evaluate neuronal differentiation by determining whole-cell sodium current densities. However, only one out of 29 MMC-treated and none of 45 untreated neuron-like mESCs showed inward voltage-activated currents (Figure 1C). We then analyzed the dopaminergic phenotype by measuring neurotransmitter release. Differentiated cultures that had been pre-treated with MMC spontaneously released four times more dopamine than untreated cells (untreated: 4.16 ± 0.47 and MMC-treated: 16.59 ± 2.13 pg/µg protein over 48 h. Figure 1D). DA release evoked by high KCl also increased about five times after MMC treatment (untreated: 0.69 ± 0.10 and MMC-treated: 3.55 ± 1.50 pg/µg protein after 15 min incubation in 60 mM KCl. Figure 1D). These results show that, although the differentiating mESCs were not electrically mature at 14 days, 1 µg/mL MMC pre-treatment promoted a functional dopaminergic phenotype, increasing both spontaneous and stimulated neurotransmitter release.

### Pre-treatment with 1 µg/mL Mitomycin C Prevents Teratoma Formation by mESCs Grafted into Nude Mice

Grafts of undifferentiated mESCs are prone to develop into tumors, particularly teratomas, as expected from their pluripotency (Fong et al., 2010). Standard protocols to assess tumorigenesis consist of injecting cells into the sub-renal capsule, the liver, the myocardium, the brain, or into subcutaneous or intramuscular sites. We chose to administer intramuscular injections into the hindlimb muscles because this is a straightforward strategy known to be efficient for assessing pluripotency capacity, besides being less harmful to the animal. Additionally, this strategy allowed us to use each animal as its own control by injecting control mESCs or MMC-treated cells in each limb.

Twelve weeks after injection of 500,000 cells, all left hind paws that received control cells developed prominent tumors, revealing the proliferative capacity of mESCs (Figure 2A). *In vivo* MRI confirmed formation of unilateral heterogeneous tumors (Figure 2B). Tumors were also evidenced in transverse images before (Figure 2C) and after gadolinium-contrast enhancement (Figure 2C). Histopathological analysis (Figure 2D) revealed that all tumors were mature teratomas containing ectodermal- (Figure 2E) as well as endodermal- (Figure 2F) and mesodermal-derived (Figure 2G) tissue, further confirming the pluripotency of non-MMC-treated mESCs. Remarkably, the same number of MMC-treated mESCs did not induce any tumor in the right paws of the same animals (*n* = 6) (Figures 2A–C). These results indicate that *in vitro* pre-incubation with 1 µg/mL MMC for 12 h completely blocked further mitotic activity of grafted mESCs *in vivo*.

### Striatal Graft with Mitomycin C-Treated mESCs Restores Motor Function in Hemi-Parkinsonian Mice Without Teratoma Induction

To test for potentially beneficial effects of mESC transplantation into a dopamine-deficient hemisphere and to investigate whether MMC could prevent tumor formation in the living brain, we injected MMC-treated pluripotent mESCs into the striatum of mice that had been previously lesioned by 6-OHDA. Every other week, transplanted animals were weighed, underwent behavioral testing for motor function, and were submitted to MRI scans to check for possible formation of brain tumors and to assess blood–brain barrier integrity. As expected, unilateral striatal lesion consistently resulted in severe motor dysfunction, but was not lethal *per se*. However, animals that received intrastriatal transplants of 500,000 untreated mESCs died between 3 and 7 weeks later (Figure 3A). In all animals from this group, intracerebral tumors were revealed by 7 Tesla MRI scans as early as 05 days after mESC transplant (Figure 3B). Importantly, contrast in intracerebral tumor images was significantly enhanced after gadolinium injection, further indicating a disruption of the integrity of the blood–brain barrier in these animals (Figure 3B, right). Post-mortem histological analysis with HE staining of all 8 transplanted hemispheres confirmed the presence of solid tumors after transplantation with untreated mESCs. In addition, immunohistochemistry for TH (Figures 3E–G) and computer-microscope reconstruction (Figure 3D) revealed a population of dopaminergic cell bodies restricted to the injected striatum (dots indicate cell bodies positive for TH-immunostaining). In contrast, all animals receiving MMC-treated mESCs survived until the end of the observation period of 12 weeks post-transplant (*n* = 12 mice; Figure 3A), and no tumors could be detected in contrast-enhanced MRI scans (Figure 3C) or through histopathological investigation. Four additional MMC-mESC transplanted mice were monitored for as long as 60 weeks with no signs of pathology.

All animals lesioned by unilateral intrastriatal 6-OHDA injection developed motor signs of dopamine deficiency that were stable after four weeks, as previously described (Iancu et al., 2005). At this time point, the rate of apomorphine-induced contralateral full-body rotations ranged from 15 to 20 rpm amongst all 6-OHDA lesioned animals. For ease of comparison, rotation rates for each experimental group were normalized relative to the average rate measured on the week of transplantation (week 0). 6-OHDA-lesioned mice injected with culture medium (6-OHDA + medium, *n* = 8) maintained a steady behavioral response to apomorphine, displaying 0.98 ± 0.19 relative rpm at 20 weeks after the lesion (Figure 4A). Transplantation with untreated mESCs (6-OHDA + mESCs, *n* = 8) led to a marked reduction in the rate of apomorphine-induced rotations, before generating brain tumors that eventually killed the animals. Mice transplanted with MMC-treated mESCs (6-OHDA + MMC-mESCs, *n* = 12) displayed a slower but steady decrease in the number of apomorphine-induced rotations, reaching 0.13 ± 0.11 relative rpm 12 weeks after transplant (Figure 4A). Already at 2 weeks after transplant, the difference relative to 6-OHDA + medium control animals was significant (*p* < 0.001, Bonferroni test in RM ANOVA).

Long-term motor improvements resulting from MMC-treated mESC were further assessed 12 weeks after transplantation using open field, grip strength, beam walking, and cylinder tests. To assess the extent of recovery from the initial lesion, behavioral scores were compared across 6-OHDA + medium control, 6-OHDA + MMC-treated mESC, and healthy, non-lesioned, non-transplanted, aged-matched mice (normal group, *n* = 8). Locomotor activity in the open field arena (measured as the distance covered by the animal over 5 min) was reduced by 39% on average in 6-OHDA + medium animals compared to healthy normal mice (normal: 2415 ± 373 cm; 6-OHDA + medium: 1468 ± 187 cm), but only by 17% in 6-OHDA + MMC-treated mESC animals (2003 ± 196 cm; Figure 4D). In the beam walking test for fine motor coordination and balance, 6-OHDA + medium mice slipped approximately three times as often (6.5 ± 1.8 slips, Figure 4B) and took twice the time to cross the 50 cm beam (11.3 ± 0.8 s, Figure 4C) as did healthy normal mice (2.0 ± 1.1 slips and 5.9 ± 1.5 s crossing time, respectively). Animals injected with 6-OHDA + MMC-treated mESCs displayed a significant improvement, so that both measures approached normal levels 12 weeks after transplant (2.6 ± 0.9 slips and 7.6 ± 1.9 s crossing time). None of the animals tested fell while crossing the beam. Forelimb grip strength was markedly reduced in 6-OHDA + medium mice (46.8 ± 13.1 g) as compared with healthy normal mice (123.0 ± 21.0 g), but 6-OHDA + MMC-mESC transplanted animals completely recovered muscle strength (146.9 ± 36.7 g; Figure 4E). Finally, 6-OHDA + MMC-treated mESC transplanted hemi-parkinsonian mice behaved almost like healthy mice regarding symmetric forelimb use in the cylinder test (normal: 66.3 ± 7.4% and MMC-mESC: 71.1 ± 13.6% double contacts, respectively; Figure 4F), whereas 6-OHDA + medium mice made significantly fewer symmetric contacts with the cylinder wall (16.1 ± 5.4%), indicating marked motor asymmetry. Our data show that MMC-treated mESC transplant induced considerable recovery of motor function in a mouse model of Parkinsonism, without any signs of tumor formation for up to 15 months following transplant.

## Discussion

In the present study, we show that transplantation of mESCs, pre-treated with the FDA-approved chemotherapeutic agent MMC, in the 6-OHDA-lesioned mouse model of Parkinson’s disease resulted in significant improvement in motor function and reduced akinesia without tumor formation. Furthermore, we show that halting mitotic activity of undifferentiated mESCs induced a four-fold increase in the release of dopamine after in vitro differentiation (Figure 5).

A central concern related to the therapeutic use of pluripotent stem cells in PD is the eradication of non-dopaminergic neurons and, especially, of residual stem cells with tumorigenic potential (Sykova and Forostyak, 2013). Additionally, most available studies use time-consuming protocols requiring multiple steps for generation of a limited number of DA neurons *in vitro* (Lindvall, 2012). Also, it is not fully understood whether *in vitro*-derived neurons are able to maintain their functional properties after *in vivo* transplantation (Park et al., 2006). All these caveats limit translation of this approach to the clinical practice. As shown in this study, the use of fully undifferentiated mESCs may represent a novel alternative.

Undifferentiated mESCs are easier to maintain than differentiated cultures; they are less expensive to cultivate and easier to obtain on a large scale. We have recently developed a method to cultivate human embryonic stem cells on suspended beads combined with the use of stirred microcarrier systems, with an optimized xeno-free culture medium (Marinho et al., 2013). Using this protocol, it is possible to produce over 160 million pluripotent cells per week, providing a step forward towards therapeutic use of human embryonic stem cells.

Much attention has been given to the benefits of using autologous induced pluripotent stem cells (iPSCs) instead of embryonic stem cells in preclinical and clinical studies. However, Araki et al. (2013) have shown that iPSCs and embryonic stem cells share similar limited immunogenicity. Importantly, undifferentiated embryonic stem cells are less immunogenic than their differentiated derivatives (Bonde and Zavazava, 2006).

Recently, several protocols for cell purification have been proposed as alternatives to eliminate potential tumorigenic cells (Kahan et al., 2011; Sundberg et al., 2013) or to obtain highly enriched populations of dopaminergic neurons (Pruszak et al., 2007; Bernstein and Hyun, 2012), without the need for genetic manipulation of cells. Thus, magnetic (MACS) or fluorescence-activated cell sorting (FACS), associated with the use of antibodies against specific cell surface marker antigens (generally, clusters of differentiation markers), have been incorporated as important tools for transplantation studies. However, cell sorting procedures are not fully efficient, besides the fact that such multi-step techniques may compromise the viability of fragile neuronal cell types (Emre et al., 2010).

MMC is a DNA-alkylating agent that irreversibly blocks cell division, inducing arrest mainly in the S phase of the cycle (Witte and Bradke, 2008). Acting as a DNA crosslinking agent, MMC could potentially be genotoxic to stem cells. Vinoth et al. (2008) investigated how genotoxic stress caused by MMC affects human embryonic stem cells as compared to a somatic human fetal lung fibroblast cell line, and found a 10% increase in the number of aberrant somatic cells vs. a 5% increase in human embryonic stem cells, indicating that the latter are more resistant to MMC-induced damage.

Due to its cytotoxic activity, we tested the impact of MMC pre-treatment on the viability and differentiation potential of mESCs. Most mESCs pre-treated with MMC and co-cultured onto a feeder layer of meningeal cells for neuronal induction exhibited typical neuronal morphology and expressed β-tubulin class III and TH. Interestingly, pre-treated cells released five times more dopamine, as compared to non-treated ones. These results indicate that MMC treatment promotes mESC differentiation towards a functional dopaminergic phenotype. Using a different cell type and experimental approach, Felfly et al. (2011) showed that MMC alone trigger the expression of several differentiation genes in murine neural stem cells.

To investigate the intrinsic tumorigenic capacity of pluripotent stem cells, we tested if MMC treatment blocks teratoma formation. Results with nude mice showed that pre-treatment of cells with 1 µg/mL MMC for 12 hours suppressed the tumorigenic capacity of transplanted mESCs after least 12 weeks. Untreated mESCs transplanted in the opposite hindlimbs of the same animals generated large teratomas.

As might be expected, mice injected in the striatum with non-treated mESCs developed tumors and exhibited blood–brain barrier disruption, as assessed by periodic MRI scans, which may explain why none of those animals survived more than seven weeks after transplantation. Nonetheless, despite the presence of intracerebral teratomas, all animals that received non-treated mESCs performed better on the apomorphine-induced rotation test. We suggest that this effect may be due to the differentiation of transplanted mESCs into dopaminergic neurons *in vivo*. In support of this notion, Felfly et al. (2011) described grafts able to generate DA neurons, which were surrounded by glial cells from the host, suggesting that neural differentiation of transplanted cells may be influenced by glial signaling *in vivo*. Moreover, mesencephalic neuroepithelial stem cells differentiated into tyrosine hydroxylase-positive neurons 3 times more efficient when transplanted into the brain of PD brains than in controls, which could also be explained by the presence of environmental cues (Mine et al., 2009). Finally, Takagi et al. (2005) demonstrated that FGF20, preferentially expressed by cells within the substantia nigra, increases the differentiation of DA neurons from ESC-derived neurospheres in monkeys.

Transplantation of MMC-treated mESCs in the brain of 6-OHDA lesioned animals significantly reduced the rate of apomorphine-induced rotations and nearly normalized motor performance at 12 weeks post-transplant. However, the beneficial effect of transplantation was somewhat slower in these animals than in mice transplanted with non-treated cells. This lies in apparent contradiction with our in vitro data showing higher spontaneous and stimulated dopamine release from differentiated MMC-treated mESCs. Nevertheless, untreated mESCs obviously proliferated faster, maybe generating more dopaminergic cells than MMC-treated mESCs. It will be of interest to assess whether survival, differentiation, and tissue integration are directly affected by prior MMC treatment such as proliferation.

In conclusion, results presented here show that MMC treatment allowed pluripotent stem cells to restore motor function without forming tumors for as long as 15 months in mice, suggesting that MMC-treated undifferentiated embryonic stem cells should be further investigated as a safe and effective strategy for the treatment of Parkinson’s disease.

## Conflict of Interest Statement

The authors declare that the research was conducted in the absence of any commercial or financial relationships that could be construed as a potential conflict of interest.

## Footnotes

^1^www.osirix-viewer.com
